# Hypothetical granulin-like molecule from *Fasciola hepatica* identified by bioinformatics analysis

**DOI:** 10.1186/s40064-016-2443-7

**Published:** 2016-06-17

**Authors:** Claudia Machicado, Luis A. Marcos, Mirko Zimic

**Affiliations:** Bioinformatics Laboratory, Department of Cellular and Molecular Sciences, Faculty of Sciences and Philosophy, Universidad Peruana Cayetano Heredia, Av. Honorio Delgado 430, Lima 31, Peru; Institute for Biocomputation and Physics of Complex Systems, University of Zaragoza, Mariano Esquillo, Edificio I+D, 50018 Saragossa, Spain; Division of Infectious Diseases, Department of Internal Medicine, Stony Brook University, Stony Brook, 100 Nicolls Road, Stony Brook, NY 11790 USA; Instituto de Medicina Tropical Alexander von Humboldt, Universidad Peruana Cayetano Heredia, Av. Honorio Delgado 262, Lima 31, Peru

**Keywords:** Fasciola, Granulin, Helminth, Homology, Proliferation, Carcinogenesis

## Abstract

*Fasciola hepatica* is considered an emergent human pathogen, causing liver fibrosis or cirrhosis, conditions that are known to be direct causes of cancer. Some parasites have been categorized by WHO as carcinogenic agents such as *Opisthorchis viverrini*, a relative of *F. hepatica*. Although these two parasites are from the same class (Trematoda), the role of *F. hepatica* in carcinogenesis is unclear. We hypothesized that *F. hepatica* might share some features with *O. viverrini* and to be responsible to induce proliferation of host cells. We analyzed the recently released genome of *F. hepatica* looking for a gene coding a granulin-like growth factor, a protein secreted by *O. viverrini* (*Ov*-GRN-1), which is a potent stimulator of proliferation of host cells. Using computational biology tools, we identified a granulin-like molecule in *F. hepatica,* here termed FhGLM, which has high sequence identity level to *Ov*-GRN-1 and human progranulin. We found evidence of an upstream promoter compatible with the expression of FhGLM. The FhGLM architecture showed to have five granulin domains, one of them, the domain 3, was homologue to *Ov*-GRN-1 and human GRNC. The structure of the FhGLM granulin domain 3 resulted to have the overall folding of its homologue the human GRNC. Our findings show the presence of a homologue of a potent modulator of cell growth in *F. hepatica* that might have, as other granulins, a proliferative action on host cells during fascioliasis. Future experimental assays to demonstrate the presence of FhGLM in *F. hepatica* are needed to confirm our hypothesis.

## Background

Fascioliasis, a zoonotic parasitic disease caused by either *F. hepatica* or *Fasciola gigantica,* is a major public health problem in many tropical and subtropical regions. Recent reports have estimated that between 2 and 17 million people are infected, and 180 million people are at risk of infection; prevalence is particularly high in the Andean highlands of Peru, Ecuador, and Bolivia (Gonzalez et al. 2011; Fürst et al. 2012). Nowadays fascioliasis is considered the most widespread trematode disease affecting grazing animals around the world and its causing agent, Fasciola, has been recognized by the World Health Organization as an emergent human pathogen.

Chronic infection by *F. hepatica*, as occurs in other liver flukes, produces physical tissue damage induced by the feeding activities. The suckers of the fluke hook into the biliary epithelia, damaging the bile ducts, even in the early infection. As the flukes mature, the lesions enlarge and ulcerate (hemobilia). As demonstrated in *O. viverrini*, the biliary damage predispose to develop cancer (Sripa et al. 2007). This mechanism of mechanical damage is present in other cancer-causing pathogens such as *Schistosoma haematobium* (Rosin et al. 1994a, b) and *Helicobacter pylori* (Niwa et al. 2010). In contrast, there is another mechanism present in pathogens that are known to induce which is from the release of toxic/carcinogenic parasite excretory/secretory (ES) molecules (Thuwajit et al. 2004; Chang et al. 2014; Wang et al. 2014; Daorueang et al. 2012). This latter cancer-promoting process has been well characterized in *O. viverrini*, which produces and secretes multiple ES molecules that are immunogenic but also toxic to host cells. One of these ES molecules is the granulin-like growth factor produced by *O. viverrini* termed *Ov*-GRN-1, which has been demonstrated to promote mammalian cell proliferation (Smout et al. 2009; Young et al. 2014; Smout et al. 2011).

Granulins are a group of highly conserved growth factors that have been described from a variety of organisms spanning the metazoan (Hanington et al. 2008). Granulins are a family of secreted, glycosylated peptides that are cleaved from a single precursor protein, known as pro-granulin (PGRN) pro-epithelin, with one or more repeats of a highly conserved 12-cysteine granulin/epithelin motif (Ong and Bateman 2003). Complete cleavage of full length PGRN results in active granulin peptides. In mammals, granulins are derived from a larger pro-granulin (PGRN) that produces 7 active peptides (GRN 1-7 and paragranulin) of approximately 6 kDa in size (De Muynck and Van Damme 2011; Bhandari et al. 1992; Plowman et al. 1992). Both the intact precursor and the single granulin are able to modulate cell growth (Culouscou et al. 1993; Shoyab et al. 1990; Zhou et al. 1993). However, different members of the granulin protein family may act as inhibitors, stimulators, or have dual actions on cell growth. For instance, whereas human GRN-4 (also known as GRNA) demonstrated to be a potent growth inhibition of a breast cancer cell line, human GRN-2 (also known as human GRNF) was stimulatory (Tolkatchev et al. 2008).

Human progranulin (PGRN) has been associated with many aggressive cancers such as CCA and its overexpression is related to tumor growth, angiogenesis and resistance to apoptosis (Demorrow 2013). Of note, the oncogenic parasite *O. viverrini* releases the granulin (*Ov*-GRN-1) that has been shown to be mitogenic at very low concentrations (Smout et al. 2009). Most recently, a novel single-domain ES granulin (Ov-GRN-2) was identified in *O. viverrini* but its function remains unknown (Young et al. 2014).

Given the close phylogenetic relationship between liver flukes such as *O. viverrini* and *F. hepatica*, it would be possible that they may have similar biological products (i.e. growth factors). The discovery of growth-factor like molecules in *F. hepatica* may provide vital information and insights into the fundamental biology of this parasite, identify related pathways linked to fluke-host interactions and predict interactions from host factors into the disease. The aim of this study is to identify a potential growth-factor, topologically similar to both *Ov*-GRN-1 and PGRN, in *F. hepatica* genome by using computational biology tools.

## Results

### Identification of *Ov*-GRN-1 homologue in *F. hepatica* genome

**Fig. 1:**
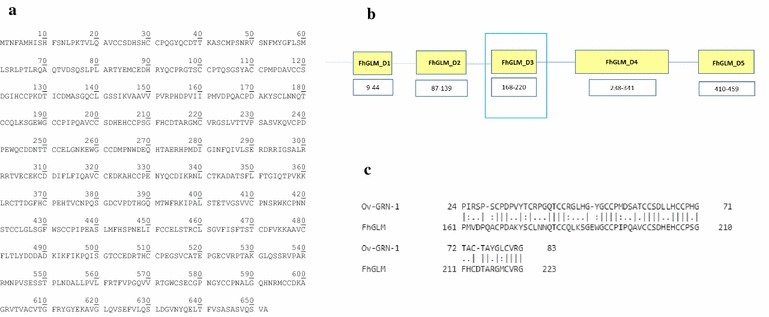
Aminoacid sequence and architecture of FhGLM. **a** Aminoacid sequence of the GRN candidate (FhGLM) identified from the *F. hepatica* genome. **b** Grn domains predicted by SMART for FhGLM (Fh-GLM-D1 to Fh-GLM-D5) are shown in *yellow boxes* with their sequence numbering below each one. Maximum identity region of FhGLM with Ov-GRN-1 is squared by a *Green box*. **c** Pairwise sequence alignment of the FhGLM and *Ov*-GRN-1 (Swissprot:ACJ83119) showing the maximum identity region in Fh-GLM-D3; identical residues are linked by *sticks* and similar residues are linked by *dots*

To identify potential *F. hepatica* GRN-like sequences we screened for closely related helminth parasite *O. viverrini* granulin (*Ov*-GRN-1) sequence in the *F. hepatica* genome available in the WormBase ParaSite website under the code PRJEB6687. The Blastp search returned 9 hits for the *Ov*-GRN-1 query. All of them were originated from the same scaffold numbered 891. The two top entries were selected because of their lower E-values cut-off resulting as of 1.2E^−14^ and 7.0E^−11^, respectively. Both entries corresponded to two different regions within the deduced protein of scaffold 891 whose transcript was identified as to BN1106_s891B000441.mRNA-1 (a product of the gene BN1106_s891B000441), which has 1959 bp and 9 exons. An upstream promoter was identified by Neural Network Promoter Prediction, which is compatible with the expression of this gene. The homologue protein of *Ov*-GRN-1 identified in the *F. hepatica* genome (translation ID BN1106_s891B000441.mRNA-1 in the WormBase ParaSite) has 652 residues, a molecular weight of 71.48 kDa and pI of 6.02 (Fig. 1a). The *Ov*-GRN-1 aligned with two adjacent regions over the *F. hepatica* granulin-like molecule; the first region located from residues 70-146 (Domain 2) and the second on located from residues 161-223 (Domain 3). Domain 2 was 41 % identical to *Ov*-GRN-1 with a significant E-value 7.0E^−11^ while Domain 3 was 54 % identical to *Ov*-GRN-1 with a significant E-value 1.2E^−14^. Subsequent Blastp searching against the bovine genome showed that scaffold 891 (hereafter referred to as *F. hepatica* GRN Like Molecule, FhGLM) displayed 28 % identity to bovine granulin isoform X2 (GenBank:XP_010814706.1), a predicted GRN protein. Therefore, FhGLM was identified as possibly encoding proteins containing the GRN conserved domain.

### FhGLM features

To further confirm the presence of the hypothetical GRN-like domains, FhGLM was individually searched for the conserved GRN domain. Typically, the GRN domain is constituted by 12 conserved cysteins arranged into four cysteine pairs and flanked by two single cysteines at both the amino and carboxy terminal (Bateman et al. 1990). The structural architecture of the FhGLM was similar to the granulin domain IPR000118 deposited in InterPro as shown in Fig. 1b. The putative FhGLM has five granulin domains as predicted by SMART, PFAM and Prosite patterns. Its length suggests that FhGLM is a precursor composed of 652 residues distributed in 5 different GRNs, numerically designated from 1 (Fh-GLM-D1) to 5 (Fh-GLM-D5) based on the order in the sequence (Fig. 1b). Compared to *Ov*-GRN-1, FhGLM was significantly longer (550 residues more than *Ov*-GRN-1) and it looks like a multi-homodomain GRN protein in contrast with *Ov*-GRN-1 that has a single GRN domain behind a secretory signal peptide (Smout et al. 2009). One granulin domain in FhGLM, here termed Fh-GLM-D3 (from residues 168-220), was homologue to *Ov*-GRN-1 (from residues 24-83) resulting in 54 % identity (Fig. 1c). Both *Ov*-GRN-1 and *Fh*-GLM-D3 have granulin domain cores composed by 12 cysteins over its sequence (Fig. 1c). When compared to the human GRN precursor or human PGRN, (Swiss-Prot:NP_002078.1), FhGLM is longer (109 residues more than human PGRN) and both are multi-homodomain GRN proteins. The Fh-GLM-D3 resulted homologue to the human GRN-5 or human GRNC (from residues 364-417 in the human PGRN) showing 67 % identity. Glycosylation sites were predicted in the FhGLM. While no N-linked glycosylation site was predicted, four O-linked sites were predicted at Thr-2, Thr-40, Thr-98, and Thr-103. Different from *Ov*-GRN-1 and human PGRN, the FhGLM showed no N-terminal signal peptide but it was predicted to be a secretory protein. This finding suggests that the FhGLM may be secreted by an ER/Golgi-independent mechanism.

Other protein features were evaluated in the *Ov*-GRN-1, Fh-GLM-D3 and human GRNC as shown in Table 1. Fh-GLM-D3 has an intermediate composition of negatively charged residues Asp and Glu (7.8 %) when compared to human GRN (11.2 %) and *Ov*-GRN-1 (4.9 %). The FhGLM-D3 has a composition of positively residues Arg and Lys (5.2 %) comparable to *Ov*-GRN-1 (7.8 %) but markedly different to human GRNC, which has no Arg and Lys residues in its composition. The content of Cys residues is similar in the Fh-GLM-D3 (22.6 %) compared to human GRNC (22.2 %), which is higher than content of cysteins in *Ov*-GRN-1 (12 %). The theoretical point isoelectric (pI) of Fh-GLM-D3 (5.76) and human GRNC (4.01) were notably lower than *Ov*-GRN-1 (8.29). In accordance to those findings, the theoretical charges at pH 7 showed that the Fh-GLM-D3 has an acidic profile (charge of −1.7) similar to the human GRNC (charge of −6) but opposed to *Ov*-GRN-1 (charge of 3.8).

**Table 1:** Comparison of biological features of *Ov*-GRN-1, hGRNC (human) and Fh-GLM-D3

Protein name	Length (aa)	MW (Da)	pI	Charge	Cys (%)	Asp + Glu (%)	Arg + Lys (%)
*Fh*-GLM-D3	52	5742	5.76	−1.7	22.6	7.8	5.2
hGRNC	54	5671	4.01	−6	22.2	11.2	0
*Ov*-GRN-1	102	91,596	8.29	3.8	12	4.9	7.8

*MW* molecular weight, *pI* point isoelectric, *Cys* cystein residues, *Asp* *+* *Glu* total content of aspartic and glutamic residues, *Arg* *+* *Lys* total content of arginin and lysin residues

Comparative analysis with other GRN members showed that FhGLM is substantially longer than *Ov*-GRN-1 (102 residues) and *Brugia malayi* GRN (77 residues). The structural architecture in the two latter granulins are constituted by one GRN domain only. At the contrary FhGLM was predicted to have 5 GRN domains similarly to other organisms considered in our analysis and that present more than one GRN domain, including helminths *Schistosoma japonicum* and *S. haematobium, Clonorchis sinensis, Ascaris suum, Haemonchus contortus, Caenorhabditis elegans, Strongyloides ratti, Echinococcus Granulosus* and vertebrates including *Mus musculus, Homo sapiens, Bos taurus.*

### Phylogenetic analysis

To examine sequence features of the candidate GRN family member, we performed a multiple sequence alignment of known GRN proteins from both mammalian hosts and helminths. Bovine and human PGRN were included as reference sequences for analysis.

**Fig. 2:**
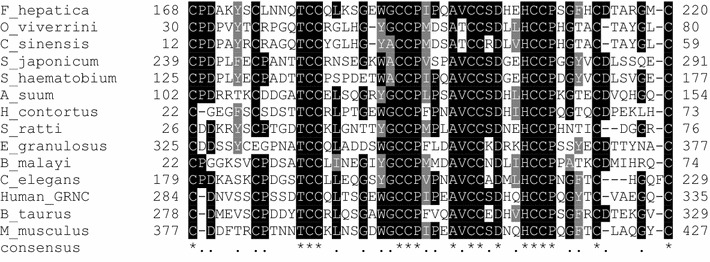
Multiple sequence alignment of the deduced amino acid sequence of the Fh-GLM-D3 from *F. hepatica* and known GRN proteins. Conserved cysteines among GRN family are shown in *black boxes*. The consensus sequence is shown in the *bottom*. Accession numbers for GRN sequences used in this analysis are: *O. viverrini* (Swiss-Prot:ACJ83119.1), *F. hepatica* Fh-GLM-D3 (WormBase ParaSite:BN1106_s891B000441.mRNA-1), *C. sinensis* (GenBank:AT006891), *S. japonicum* (Swiss-Prot:AAX25968.2), *S. haematobium* (Swiss-Prot:XP_012796138.1), *A. suum* (Swiss-Prot:U1MDS0), *H. contortus* (Swiss-Prot:U6P5A2), *S. ratti* (Swiss-Prot:A0A090LCJ6), *E. granulosus* (Swiss-Prot:CDS24124.1), *B. malayi* (Swiss-Prot:CDQ02690.1), *C. elegans* (Swiss-Prot:NP_492981.1), *H. sapiens* hGRNC (Swiss-Prot:NP_002078.1), *M. musculus* (Swiss-Prot:NP_032201.2), *B. taurus* (GenBank:XP_010814706.1). The image was obtained with the BoxShade tool at Expasy web server (http://www.ch.embnet.org/software/BOX_form.html)

The alignment of Fh-GLM-D3 with other GRN family members showed that all GRN family members, and also Fh-GLM-D3, contained 12 conserved Cys residues arranged into four Cys pairs and flanked by two single Cys at both the amino and carboxyl termini (Fig. 2). The conservation pattern observed in the GRN family analyzed suggests the relevance of maintaining a number and types of residues in the granulin core, which is most likely to preserve its function (Fig. 2).

**Fig. 3:**
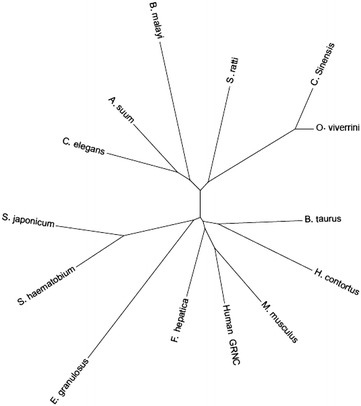
Phylogenetic relationships of mammalian GRN and helminth GRN homologues. The *circular phylogenetic tree* represents the relationship of the sequences of mammalian GRN family members (human, mouse and bovine) and GRN homologues of helminths

The evolutionary relationship between the *Ov*-GRN-1 homologue found in *F. hepatica*, Fh-GLM-D3, and other members of GRN superfamily from helminths and mammalian hosts was investigated by multiple alignment and subsequent phylogenetic tree construction by Seaview version 4 software (Galtier et al. 1996; Gouy et al. 2010). Phylogenetic analysis of the conserved GRN domain cores was carried out on 14 sequences of helminth and mammal GRN proteins (Fig. 3). Human GRNC was included since it was the template for structure modelling of Fh-GLM-D3. The phylogenetic tree displayed two subfamilies of granulin; one of them contained the GRN from the mammalian hosts (human GRNC, *M. musculus,* human GRNC, and *B. taurus*) very closely with *F. hepatica* and the nematode *H. contortus* whereas the cestode *E. granulosus* was grouped closely with the blood flukes (Schistosoma) in a separate clade. The other subfamily grouped the trematodes *O. viverrini* and *C. sinensis* with the nematode *S. ratti* in a clade whereas GRNs from the nematodes *A. suum, C. elegans and B. malayi* were grouped in a separate clade.

### FhGLM predicted structure

Both the complete FhGLM (652 residues) and the Fh-GLM-D3 (52 residues) sequences were submitted to Phyre2 to build the corresponding structures. The complete FhGLM structure was constructed as follows: 58 % of residues were modelled at >90 % confidence using multiple-templates and applying both homology modeling and ab initio Ping 1.0 approach (Jefferys et al. 2010). The predicted FhGLM is composed by 11 β-sheets, no α-helix is detected. The confidence score of this model was 90 % indicating that the model of the complete FhGLM was not very precise. The covalent geometry of the complete modeled structure resulted in 72.6 % of residues in favored regions and allowed of the Ramachandran Plot and ProSA Z-score of −3.38 (Wiederstein and Sippl 2007; Sippl 1993). The model contained an α-helix and 11 antiparallel β-strands, 4 of which are small ones. This model included long loop regions and this was also seen in the Ramachandran Plot (Ramachandran et al. 1963).

**Fig. 4:**
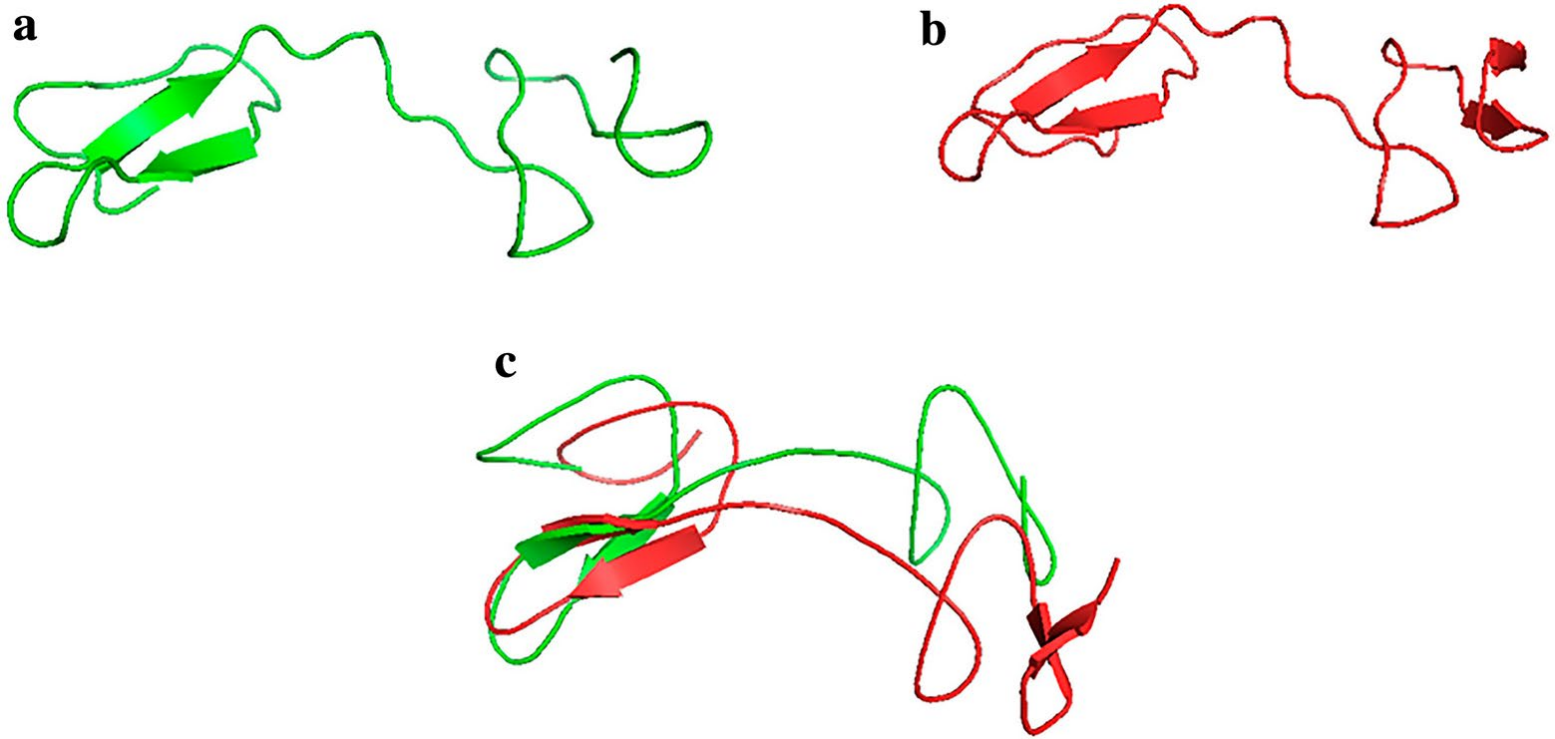
3D structure of predicted Fh-GLM-D3 from Phyre2 by homology search modelling based on template 2JYT (human GRNC). **a** Fh-GLM-D3. **b** Human GRNC (PD: 2JYT). **c** Overlay showing the Fh-GLM-D3 in green and the human GRNC in *red*. *Source*: PyMOL

In contrast, Fh-GLM-D3 was constructed taking the human GRNC NMR structure (PDB: 2JYE) as template, with 99.2 % confidence (Fig. 4). The high confidence score of the Fh-GLM-D3 structure indicated that the model is correct and that Fh-GLM-D3 and human GRNC are real homologs, which is supported by the considerable identity level (67 %). The predicted structure of Fh-GLM-D3 consists of 52 residues arranged in a N-terminal stack of two β-hairpins and no α-helix present in its structure (Fig. 4). Identical folding has the human GRNC used as template for modeling which is a β-strand protein, composed of 54 residues, with a well-defined N-terminal stack of two β-hairpins and C-terminal of two short antiparallel β-strands. The Fh-GLM-D3 had 90 % of residues in favored and allowed regions of the Ramachandran Plot and ProSA Z-score of −5.41. The template structure (PDB: 2JYT) had 94 % of residues in most favored regions of the Ramachandran Plot and ProSA Z-score of −4.75. The high similarity between these overall quality parameters confirmed the good model quality. Both the Fh-GLM-D3 and human GRNC included many loop regions in their structures. This can also be seen in the Ramachandran Plot where 10 and 6 % residues of these proteins were present in non-regular regions (i.e. out of β-strands and helices), respectively. The disorder propensity predictions in Fh-GLM-D3 and human GRNC done by IUPRED, a software devoted to the search of intrinsically disordered regions, predicted ordered segments both on Fh-GLM-D3 and on the human GRNC. In addition, the comparison of the secondary structures between human and Fh-GLM-D3 model has also showed that secondary structure elements including β-strands and loops were well conserved along the sequence, just few changes were observed in their length (Fig. 4).

The local model quality was assessed by calculating the knowledge-based energy, which resulted quite favorable both in the Fh-GLM-D3 and the resolved human GRNC. In contrast, the knowledge-based energy of the complete FhGLM structure resulted in positive values suggesting some problematic or erroneous parts in the modeled structure. The quality findings mentioned above agreed with the assessment conducted using ProQ2 suggesting the good quality of the Fh-GLM-D3 structure and the limitations to obtain a good quality structure of the complete FhGLM (Ray et al. 2012).

## Discussion

Granulin family members are important in normal development, wound healing, and tumorigenesis. The homologue of human GRN in *O. viverrini* (*Ov*-GRN-1) has been demonstrated to be a potent stimulator of cell proliferation and has been associated with cancer progression (Smout et al. 2009). We were interested in determining the presence of a homologue of the *Ov*-GRN-1 in *F. hepatica* genome. The GRN candidate of *F. hepatica* (FhGLM) was identified here is a homologue both of human GRN and *Ov*-GRN-1. In size and architecture terms the FhGLM, with 652 residues and 5 granulins, is similar to the human PGRN, which is composed of 543 residues and 7 GRNs. In contrast, the FhGLM is larger and has a more complex architecture than *Ov*-GRN-1, this latter is constituted of 102 residues and it contains only one GRN domain (Smout et al. 2009). The granulin 3 in FhGLM, here termed Fh-GLM-D3, has 54 % identity with *Ov*-GRN-1, an expected high similarity considering the close phylogenetic relationship of both liver flukes. The FhGLM has 29.7 % overall identity with human PGRN, a considerable similarity being non-related phylogenic organisms. In contrast, the Fh-GLM-D3 has 67 % local identity with human GRNC. Interestingly, both *Ov*-GRN-1 and human GRNC are growth factors (Smout et al. 2009; Bateman and Bennett 1998).

Primary sequence analysis of Fh-GLM-D3 using SignalP suggested that such hypothetical protein lacked predicted N-terminal signal peptides for secretion via the classical ER/Golgi pathway (Petersen et al. 2011). Such finding is in agreement with prior studies that did not describe a granulin from *F. hepatica* within the ES products. We hypothesize that Fh-GLM-D3 may be released by alternate signal-peptide independent mechanisms such as extracellular vesicles (Cwiklinski et al. 2015a; Robinson et al. 2009; Marcilla et al. 2012). In parasite such as helminths, a non-classical secretory pathway has been described in the secretion of factors such as the macrophage inhibitory factor (MIF) that involves ABC transporters (Flieger et al. 2003). Other proteins such as a secretory TGFB member that lacks signal peptide, recently described in *F. hepatica*, has not been reported in the ESP fraction or in vesicles (Japa et al. 2012). The fact that a granulin factor has not been described in ES products or extracellular vesicles may be explained by the following: (1) granulin may be one of the “unknown” or “uncharacterized” proteins reported within EVs, (2) granulin may be secreted by other mechanisms not yet described, or (3) granulin may be mostly expressed in the juvenile parasites, for which data is lacking. In summary, proteins lacking an N-terminal signal peptide, such as granulin and TGFB from *F. hepatica*, may be exported to the host interface by vesicles or other unknown mechanism (s).

The results showed that the peptide theoretically identified in FhGLM has 5 GRN conserved domains. The conservation of critical residues for protein function may be indicative of maintenance of essential functions related to proliferative action by FhGLM. The phylogenetic relationship with other GRN members demonstrated that Fh-GLM-D3 clustered within the mammal hosts group. Parasitic GRNs were clustered in separate groups from free-living organisms and mammal hosts inferring that the GRN of the parasitic worms share common function(s), which might be specific and vital to establishing a parasitic life cycle. The phylogenetic relationship of this GRN stimulating cell growth factor supports a functional protein relationships rather than taxonomic relationships as previously suggested (Smout et al. 2009).

Granulins have been demonstrated to have a function during infection with nematodes such as *Trichinella spiralis* (Wu et al. 2008). The expressions of 30 genes were identified to be up-regulated only in *T. spiralis* infection but not in *T. pseudospiralis* (Wu et al. 2008). Transcription of *T. spiralis* granulin increased 3.79-fold during host infection, which was associated to the cell cycle progression and cell motility. On the other hand, the most important parasite granulin is the *Ov*-GRN-1 which was found to be associated with cancer of bile ducts (Smout et al. 2009; Mulvenna et al. 2010). It has become clearer that *Ov*-GRN-1 is the major growth factor present in ES, at large, that induces cell proliferation and, ultimately, likely promotes cholangiocarcinogenesis. The high identity of Fh-GLM-D3 with *Ov*-GRN-1, and *T*. *spiralis* (51 %), constitutes a preliminary evidence that *F. hepatica* may have proliferative and mitogenic factors secreted to the tissue environment that might stimulate the cell proliferation, similar to *O. viverrini* and *T. spiralis*. As pro-granulins regulate cell proliferation, motility and inflammation; and they have an established role in the progression of ovarian and breast cancers (Demorrow 2013), it is plausible that this potential FhGLM may have a role in cellular transformation. Whether the infection by Fasciola in a susceptible host for tumorigenesis (i.e. patient with underlying chronic viral hepatitis or liver cirrhosis) or the infection by Fasciola itself may promote carcinogenesis is still an open question. This preliminary theoretical computational analysis sheds light on a potential protein from another parasite than Opisthorchis with a potential role in cancer. Further studies are warranted to proof this hypothesis.

## Conclusions

We found by applying theoretical approaches a granulin-like molecule in the genome of *F. hepatica* (named FhGLM) with topological features similar to its homologue in *O. viverrini*, suggesting that FhGLM might conserve its function as cell proliferative factor. Similarly, we found that the FhGLM was highly similar in topology and predicted biological features to the human progranulin, a factor that is related to tumorigenesis. In conclusion, our results suggest that *F. hepatica* genome contains a granulin gene that despite of lacking a signal peptide, it would code for a secretory protein. Its effect on proliferation of host cells either during the course of the disease or inducing directly a malignant process needs to be further investigated.

## Methods

### Searching of *Ov*-GRN1 homologue in the *F. hepatica* genome

The protein sequence corresponding to granulin-like growth factor in *O. viverrini* (*Ov*-GRN-1) was retrieved from the UniProtKB (Swiss-Prot:ACJ83119.1). Genome analysis was conducted using the putative *F. hepatica* genome produced in the laboratory of Dr. Jane Hodgkinson at University of Liverpool (Cwiklinski et al. 2015b). The *F. hepatica* draft genome was explored in the WormBase ParaSite website (http://parasite.wormbase.org/index.html). The accession number of the *F. hepatica* genome used in this work and deposited in the WormBase ParaSite was PRJEB6687. GRN like sequences were identified in the *F. hepatica* genome through a Blastp search of the draft genome contigs using protein sequence of *Ov*-GRN-1 from *O. viverrini* as query. Protein database search was activated and default terms were set for searching. An E-value cut off of 1 × 10^−4^ was used to define a significant hit. Promoter was searched by applying Neural Network Promoter Prediction (Reese 2001).

### Structural and functional features

The structural architecture of GRN family members was obtained from entry IPR000118 at the Interpro 18.0 database (Hunter et al. 2009). The *F. hepatica* granulin-like molecule was analyzed for various structural and functional features using biocomputing approaches including SignalP 4.0 (Petersen et al. 2011), Protein Predict server (Rost et al. 2004) and Balanced subCellular Localization predictor BaCelLo (Pierleoni et al. 2006). N-linked and O-linked glycosylation sites were investigated in the *F. hepatica* GRN candidate applying a prediction based on Binary profile of patterns (BPP) using GlyocoEP (Chauhan et al. 2013). Physicochemical properties including molecular mass, theoretical IP (isoelectric point), and percentage of cysteine (Cys) and charged residues (i.e. Lys, Arg, Asp, Glu) in FhGLM were predicted using the ProtParam tool of ExPaSy (http://web.expasy.org/protparam/) (Gasteiger et al. 2005). Disorder propensity prediction was performed using IUPred (Dosztányi et al. 2005a, b). Protein charge at pH 7 was estimated using Protein Calculator v 3.4 (http://protcalc.sourceforge.net/).

### Sequence analysis and phylogenetic tree

The homologue mRNA and the corresponding peptide of *Ov*-GRN-1 identified in *F. hepatica* were retrieved from the WormBase ParaSite. The amino acid sequence of *F. hepatica* GRN like-molecule was entered as query to identify homologues in other helminths using PSI Blast (Position Specific Iterated Blast) implemented in NCBI website. The amino acid sequences of GRN homologues in helminths identified by PSI Blast were retrieved from UniProtKB and GenBank. Similarly, the amino acid sequences of GRN family in mammal hosts were retrieved from UniProtKB for further analysis. The amino acid sequences of GRN homologues from helminths including *F. hepatica* candidate as well as GRN family from mammalian hosts were aligned using MUSCLE (Edgar 2004) implemented in the Seaview version 4 (Galtier et al. 1996; Gouy et al. 2010). Where multiple GRN domains were observed within one PGRN protein (e.g. vertebrates, schistosomes, *E. granulosus, C. elegans, S. ratti, H. contortus,* and *A. suum*), individual GRN domains sharing the greatest identity with Fh-GLM-D3 were selected and aligned. Core granulin domains were compared and conservation of amino acids was analyzed using Seaview version 4 (Galtier et al. 1996; Gouy et al. 2010). The phylogenetic relationship of *F. hepatica* GRN like-molecule and *Ov*-GRN-1 with other GRN family members was inferred using a neighbor joining analysis using Seaview version 4. The resulting trees were bootstrapped from 10,000 replicates to ensure accuracy.

### Structural modeling

The three-dimensional structure of FhGLM was built by searching against the Protein Data Bank (PDB) on Phyre2 (Kelley et al. 2015). Both the normal and intensive modes in Phyre2 were used to construct functional GRN domains and the full FhGLM structure, respectively. Fh-GLM-D3, which had high identity level with human GRNC (67 %), was built by homology modeling using the human GRNC [PDB: 2JYT] as the template (Tolkatchev et al. 2008). In contrast, the full FhGLM was constructed applying ab initio Poing 1.0 approach due to lack of templates appropriate for the complete sequence (Jefferys et al. 2010). To check the overall and local quality of models, ProQ2 assessment was applied within the Phyre 2 Investigator option thereby the best-scored model was selected. Pymol was used to view the homology models (http://www.pymol.org). For recognition of errors in the 3D-structure FhGLM constructed, ProSa was used (Wiederstein and Sippl 2007; Sippl 1993). The on-line tool Ramachandran plot by Rampage server was applied to the selected structures (Lovell et al. 2003).
